# Statistical Methods for Detecting Differentially Abundant Features in Clinical Metagenomic Samples

**DOI:** 10.1371/journal.pcbi.1000352

**Published:** 2009-04-10

**Authors:** James Robert White, Niranjan Nagarajan, Mihai Pop

**Affiliations:** 1 Applied Mathematics and Scientific Computation Program, Center for Bioinformatics and Computational Biology, University of Maryland, College Park, Maryland, United States of America; 2 Center for Bioinformatics and Computational Biology, University of Maryland, College Park, Maryland, United States of America; 3 Department of Computer Science, Center for Bioinformatics and Computational Biology, University of Maryland, College Park, Maryland, United States of America; King's College London, United Kingdom

## Abstract

Numerous studies are currently underway to characterize the microbial communities inhabiting our world. These studies aim to dramatically expand our understanding of the microbial biosphere and, more importantly, hope to reveal the secrets of the complex symbiotic relationship between us and our commensal bacterial microflora. An important prerequisite for such discoveries are computational tools that are able to rapidly and accurately compare large datasets generated from complex bacterial communities to identify features that distinguish them.

We present a statistical method for comparing clinical metagenomic samples from two treatment populations on the basis of count data (e.g. as obtained through sequencing) to detect differentially abundant features. Our method, *Metastats*, employs the false discovery rate to improve specificity in high-complexity environments, and separately handles sparsely-sampled features using Fisher's exact test. Under a variety of simulations, we show that *Metastats* performs well compared to previously used methods, and significantly outperforms other methods for features with sparse counts. We demonstrate the utility of our method on several datasets including a 16S rRNA survey of obese and lean human gut microbiomes, COG functional profiles of infant and mature gut microbiomes, and bacterial and viral metabolic subsystem data inferred from random sequencing of 85 metagenomes. The application of our method to the obesity dataset reveals differences between obese and lean subjects not reported in the original study. For the COG and subsystem datasets, we provide the first statistically rigorous assessment of the differences between these populations. The methods described in this paper are the first to address clinical metagenomic datasets comprising samples from multiple subjects. Our methods are robust across datasets of varied complexity and sampling level. While designed for metagenomic applications, our software can also be applied to digital gene expression studies (e.g. SAGE). A web server implementation of our methods and freely available source code can be found at http://metastats.cbcb.umd.edu/.

## Author Summary

The emerging field of metagenomics aims to understand the structure and function of microbial communities solely through DNA analysis. Current metagenomics studies comparing communities resemble large-scale clinical trials with multiple subjects from two general populations (e.g. sick and healthy). To improve analyses of this type of experimental data, we developed a statistical methodology for detecting differentially abundant features between microbial communities, that is, features that are enriched or depleted in one population versus another. We show our methods are applicable to various metagenomic data ranging from taxonomic information to functional annotations. We also provide an assessment of taxonomic differences in gut microbiota between lean and obese humans, as well as differences between the functional capacities of mature and infant gut microbiomes, and those of microbial and viral metagenomes. Our methods are the first to statistically address differential abundance in comparative metagenomics studies with multiple subjects, and we hope will give researchers a more complete picture of how exactly two environments differ.

## Introduction

The increasing availability of high-throughput, inexpensive sequencing technologies has led to the birth of a new scientific field, metagenomics, encompassing large-scale analyses of microbial communities. Broad sequencing of bacterial populations allows us a first glimpse at the many microbes that cannot be analyzed through traditional means (only ∼1% of all bacteria can be isolated and independently cultured with current methods [1]). Studies of environmental samples initially focused on targeted sequencing of individual genes, in particular the 16S subunit of ribosomal RNA [2]–[5], though more recent studies take advantage of high-throughput shotgun sequencing methods to assess not only the taxonomic composition, but also the functional capacity of a microbial community [6]–[8].

Several software tools have been developed in recent years for comparing different environments on the basis of sequence data. DOTUR [9], Libshuff [10], ∫-libshuff [11], SONs [12], MEGAN [13], UniFrac [14], and TreeClimber [15] all focus on different aspects of such an analysis. DOTUR clusters sequences into operational taxonomic units (OTUs) and provides estimates of the diversity of a microbial population thereby providing a coarse measure for comparing different communities. SONs extends DOTUR with a statistic for estimating the similarity between two environments, specifically, the fraction of OTUs shared between two communities. Libshuff and ∫-libshuff provide a hypothesis test (Cramer von Mises statistics) for deciding whether two communities are different, and TreeClimber and UniFrac frame this question in a phylogenetic context. Note that these methods aim to assess **whether**, rather than **how** two communities differ. The latter question is particularly important as we begin to analyze the contribution of the microbiome to human health. Metagenomic analysis in clinical trials will require information at individual taxonomic levels to guide future experiments and treatments. For example, we would like to identify bacteria whose presence or absence contributes to human disease and develop antibiotic or probiotic treatments. This question was first addressed by Rodriguez-Brito *et al.* [16], who use bootstrapping to estimate the p-value associated with differences between the abundance of biological subsytems. More recently, the software MEGAN of Huson *et al.* [13] provides a graphical interface that allows users to compare the taxonomic composition of different environments. Note that MEGAN is the only one among the programs mentioned above that can be applied to data other than that obtained from 16S rRNA surveys.

These tools share one common limitation — they are all designed for comparing exactly two samples — therefore have limited applicability in a clinical setting where the goal is to compare two (or more) treatment populations each comprising multiple samples. In this paper, we describe a rigorous statistical approach for detecting differentially abundant features (taxa, pathways, subsystems, etc.) between clinical metagenomic datasets. This method is applicable to both high-throughput metagenomic data and to 16S rRNA surveys. Our approach extends statistical methods originally developed for microarray analysis. Specifically, we adapt these methods to discrete count data and correct for sparse counts. Our research was motivated by the increasing focus of metagenomic projects on clinical applications (e.g. Human Microbiome Project [17]).

Note that a similar problem has been addressed in the context of digital gene expression studies (e.g. SAGE [18]). Lu *et al.* [19] employ an overdispersed log-linear model and Robinson and Smyth [20] use a negative binomial distribution in the analysis of multiple SAGE libraries. Both approaches can be applied to metagenomic datasets. We compare our tool to these prior methodologies through comprehensive simulations, and demonstrate the performance of our approach by analyzing publicly available datasets, including 16S surveys of human gut microbiota and random sequencing-based functional surveys of infant and mature gut microbiomes and microbial and viral metagenomes. The methods described in this paper have been implemented as a web server and are also available as free source-code (in R) from http://metastats.cbcb.umd.edu.

## Materials and Methods

Our approach relies on the following assumptions: (i) we are given data corresponding to two treatment populations (e.g. sick and healthy human gut communities, or individuals exposed to different treatments) each consisting of multiple individuals (or samples); (ii) for each sample we are provided with count data representing the relative abundance of specific *features* within each sample, e.g. number of 16S rRNA clones assigned to a specific taxon, or number of shotgun reads mapped to a specific biological pathway or subsystem (see below how such information can be generated using currently available software packages). Our goal is to identify individual features in such datasets that distinguish between the two populations, i.e. features whose abundance in the two populations is different. Furthermore, we develop a statistical measure of confidence in the observed differences.

**Figure 1:**
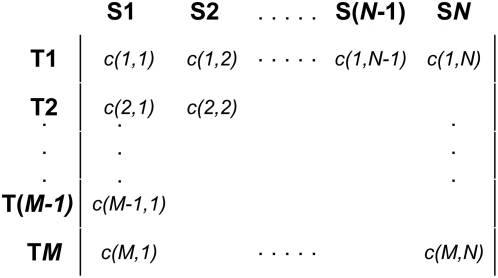
Format of the feature abundance matrix. Each row represents a specific taxon, while each column represents a subject or replicate. The frequency of the *i* ^th^ feature in the *j* ^th^ subject (*c(i,j)*) is recorded in the corresponding cell of the matrix. If there are *g* subjects in the first population, they are represented by the first *g* columns of the matrix, while the remaining columns represent subjects from the second population.

The input to our method can be represented as a *Feature Abundance Matrix* whose rows correspond to specific features, and whose columns correspond to individual metagenomic samples. The cell in the *i* ^th^ row and *j* ^th^ column is the total number of observations of feature *i* in sample *j* (Figure 1). Every distinct observation is represented only once in the matrix, i.e. overlapping features are not allowed (the rows correspond to a partition of the set of sequences).

### Data normalization

To account for different levels of sampling across multiple individuals, we convert the raw abundance measure to a fraction representing the relative contribution of each feature to each of the individuals. This results in a normalized version of the matrix described above, where the cell in the *i* ^th^ row and the *j* ^th^ column (which we shall denote *f_ij_*) is the proportion of taxon *i* observed in individual *j*. We chose this simple normalization procedure because it provides a natural representation of the count data as a relative abundance measure, however other normalization approaches can be used to ensure observed counts are comparable across samples, and we are currently evaluating several such approaches.

### Analysis of differential abundance

For each feature *i*, we compare its abundance across the two treatment populations by computing a two-sample *t* statistic. Specifically, we calculate the mean proportion 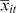, and variance 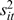 of each treatment *t* from which *n_t_* subjects (columns in the matrix) were sampled:
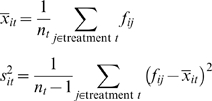
We then compute the two-sample *t* statistic:
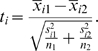
Features whose *t* statistics exceeds a specified threshold can be inferred to be differentially abundant across the two treatments (two-sided *t*-test).

### Assessing significance

The threshold for the *t* statistic is chosen such as to minimize the number of false positives (features incorrectly determined to be differentially abundant). Specifically, we try to control the p-value—the likelihood of observing a given *t* statistic by chance. Traditional analyses compute the p-value using the *t* distribution with an appropriate number of degrees of freedom. However, an implicit assumption of this procedure is that the underlying distribution is normal. We do not make this assumption, but rather estimate the null distribution of *t_i_* non-parametrically using a permutation method as described in Storey and Tibshirani [21]. This procedure, also known as the nonparametric *t*-test has been shown to provide accurate estimates of significance when the underlying distributions are non-normal [22],[23]. Specifically, we randomly permute the treatment labels of the columns of the abundance matrix and recalculate the *t* statistics. Note that the permutation maintains that there are *n_1_* replicates for treatment 1 and *n_2_* replicates for treatment 2. Repeating this procedure for *B* trials, we obtain *B* sets of *t* statistics: *t_1_^0b^*, …, *t_M_^0b^*, *b* = 1, …, *B*, where *M* is the number of rows in the matrix. For each row (feature), the p-value associated with the observed *t* statistic is calculated as the fraction of permuted tests with a *t* statistic greater than or equal to the observed *t_i_*:
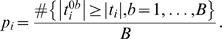
This approach is inadequate for small sample sizes in which there are a limited number of possible permutations of all columns. As a heuristic, if less than 8 subjects are used in either treatment, we pool all permuted *t* statistics together into one null distribution and estimate p-values as:
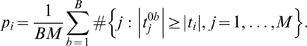



Note that the choice of 8 for the cutoff is simply heuristic based on experiments during the implementation of our method. Our approach is specifically targeted at datasets comprising multiple subjects — for small data-sets approaches such as that proposed by Rodriguez-Brito et. al. [16] might be more appropriate.

Unless explicitly stated, all experiments described below used 1000 permutations. In general, the number of permutations should be chosen as a function of the significance threshold used in the experiment. Specifically, a permutation test with *B* permutations can only estimate p-values as low as 1/*B* (in our case 10^−3^). In datasets containing many features, larger numbers of permutations are necessary to account for multiple hypothesis testing issues (further corrections for this case are discussed below). Precision of the p-value calculations is obviously improved by increasing the number of permutations used to approximate the null distribution, at a cost, however, of increased computational time. For certain distributions, small p-values can be efficiently estimated using a technique called importance sampling. Specifically, the permutation test is targeted to the tail of the distribution being estimated, leading to a reduction in the number of permutations necessary of up to 95% [24],[25]. We intend to implement such an approach in future versions of our software.

### Multiple hypothesis testing correction

For complex environments (many features/taxa/subsystems), the direct application of the *t* statistic as described can lead to large numbers of false positives. For example, choosing a p-value threshold of 0.05 would result in 50 false positives in a dataset comprising 1000 organisms. An intuitive correction involves decreasing the p-value cutoff proportional to the number of tests performed (a Bonferroni correction), thereby reducing the number of false positives. This approach, however, can be too conservative when a large number of tests are performed [21].

An alternative approach aims to control the false discovery rate (FDR), which is defined as the proportion of false positives within the set of predictions [26], in contrast to the false positive rate defined as the proportion of false positives within the entire set of tests. In this context, the significance of a test is measured by a q-value, an individual measure of the FDR for each test.

We compute the q-values using the following algorithm, based on Storey and Tibshirani [21]. This method assumes that the p-values of truly null tests are uniformly distributed, assumption that holds for the methods used in Metastats. Given an ordered list of p-values, *p*_(1)_≤*p*_(2)_≤…≤*p*_(*m*)_, (where *m* is the total number of features), and a range of values *λ* = 0, 0.01, 0.02, …, 0.90, we compute
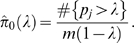
Next, we fit 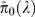 with a cubic spline with 3 degrees of freedom, which we denote 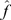, and let 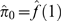. Finally, we estimate the q-value corresponding to each ordered p-value. First, 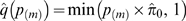. Then for *i* = *m-1*, *m-2*, *…*, *1*,
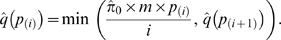
Thus, the hypothesis test with p-value 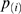 has a corresponding q-value of 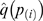. Note that this method yields conservative estimates of the true q-values, i.e. 
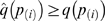
. Our software provides users with the option to use either p-value or q-value thresholds, irrespective of the complexity of the data.

### Handling sparse counts

**Figure 2:**
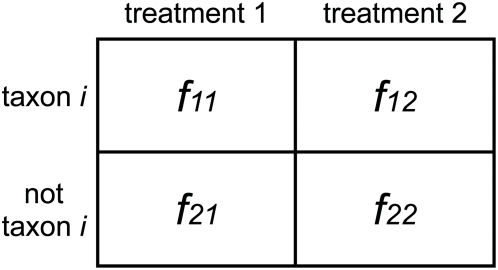
Detecting differential abundance for sparse features. A 2×2 contingency table is used in Fisher's exact test for differential abundance between rare features. *f_11_* is the number of observations of feature *i* in all individuals from treatment 1. *f_21_* is the number of observations that are not feature *i* in all individuals from treatment 1. *f_12_* and *f_22_* are similarly defined for treatment 2.

For low frequency features, e.g. low abundance taxa, the nonparametric *t*–test described above is not accurate [27]. We performed several simulations (data not shown) to determine the limitations of the nonparametric *t*-test for sparsely-sampled features. Correspondingly, our software only applies the test if the total number of observations of a feature in either population is greater than the total number of subjects in the population (i.e. the average across subjects of the number of observations for a given feature is greater than one). We compare the differential abundance of sparsely-sampled (rare) features using Fisher's exact test. Fisher's exact test models the sampling process according to a hypergeometric distribution (sampling without replacement). The frequencies of sparse features within the abundance matrix are pooled to create a 2×2 contingency table (Figure 2), which acts as input for a two-tailed test. Using the notation from Figure 2, the null hypergeometric probability of observing a 2×2 contingency table is:
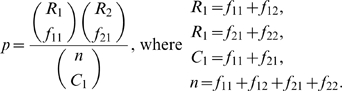



By calculating this probability for a given table, and all tables more extreme than that observed, one can calculate the exact probability of obtaining the original table by chance assuming that the null hypothesis (i.e. no differential abundance) is true [27].

Note that an alternative approach to handling sparse features is proposed in microarray literature. The Significance Analysis of Microarrays (SAM) method [28] addresses low levels of expression using a modified *t* statistic. We chose to use Fisher's exact test due to the discrete nature of our data, and because prior studies performed in the context of digital gene expression indicate Fisher's test to be effective for detection of differential abundance [29].

### Creating the Feature Abundance Matrix

The input to our method, the Feature Abundance Matrix, can be easily constructed from both 16S rRNA and random shotgun data using available software packages. Specifically for 16S taxonomic analysis, tools such as the RDP Bayesian classifier [30] and Greengenes SimRank [31] output easily-parseable information regarding the abundance of each taxonomic unit present in a sample. As a complementary, unsupervised approach, 16S sequences can be clustered with DOTUR [9] into operational taxonomic units (OTUs). Abundance data can be easily extracted from the “*.list” file detailing which sequences are members of the same OTU. Shotgun data can be functionally or taxonomically classified using MEGAN [13], CARMA [32], or MG-RAST [33]. MEGAN and CARMA are both capable of outputting lists of sequences assigned to a taxonomy or functional group. MG-RAST provides similar information for metabolic subsystems that can be downloaded as a tab-delimited file.

All data-types described above can be easily converted into a Feature Abundance Matrix suitable as input to our method. In the future we also plan to provide converters for data generated by commonly-used analysis tools.

### Data used in this paper

Human gut 16S rRNA sequences were prepared as described in Eckburg *et al.* and Ley *et al.* (2006) and are available in GenBank, accession numbers: DQ793220-DQ802819, DQ803048, DQ803139-DQ810181, DQ823640-DQ825343, AY974810-AY986384. In our experiments we assigned all 16S sequences to taxa using a naïve Bayesian classifier currently employed by the Ribosomal Database Project II (RDP) [30]. COG profiles of 13 human gut microbiomes were obtained from the supplementary material of Kurokawa *et al.* [34]. We acquired metabolic functional profiles of 85 metagenomes from the online supplementary materials of Dinsdale *et al.* (2008) (http://www.theseed.org/DinsdaleSupplementalMaterial/).

## Results

### Comparison with other statistical methods

As outlined in the introduction, statistical packages developed for the analysis of SAGE data are also applicable to metagenomic datasets. In order to validate our method, we first designed simulations and compared the results of Metastats to Student's *t*-test (with pooled variances) and two methods used for SAGE data: a log-linear model (Log-t) by Lu *et al.* [19], and a negative binomial (NB) model developed by Robinson and Smyth [20].

**Figure 3:**
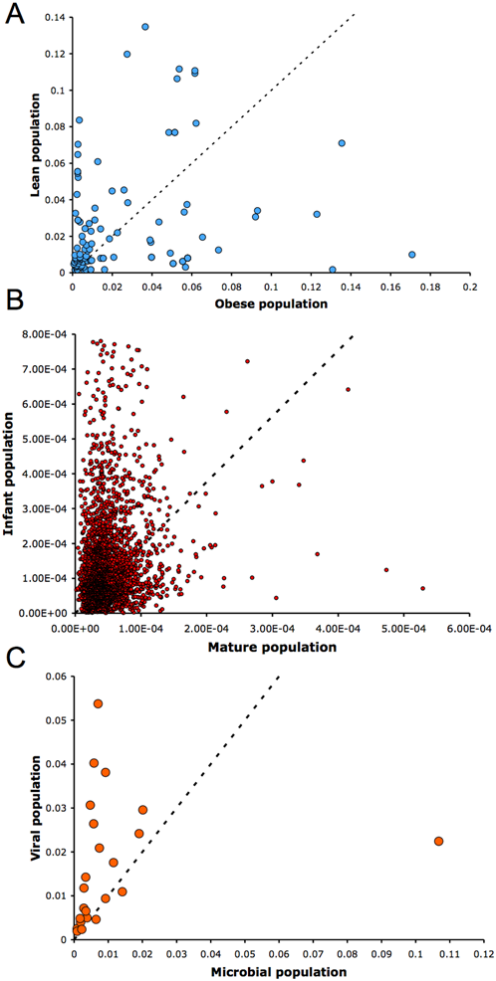
Dispersion estimates (*φ*) for three metagenomic datasets used in this study. These plots compare dispersion values between (A) obese and lean human gut taxonomic data, (B) infant and mature human gut COG assignments, and (C) microbial and viral subsystem annotations. We find a wide range of possible dispersions in this data and significant differences in dispersions between two populations.

**Figure 4:**
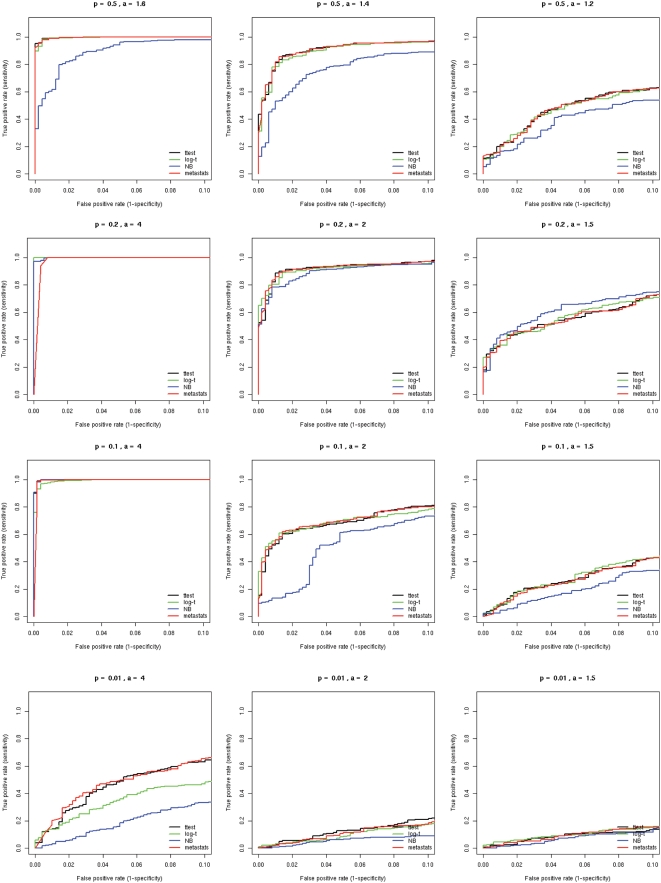
ROC curves comparing statistical methods in a simulation study. Sequences were selected from a beta-binomial distribution with variable dispersions and group mean proportions *p_1_* and *p_2_*. For each set of parameters, we simulated 1000 trials, 500 of which are generated under the null hypothesis (*p_1_* = *p_2_*), and the remainder are differentially abundant where a*p_1_ = *p_2_*. For example, p = 0.2 and a = 2 indicates features comprising 20% of the population that differ two-fold in abundance between two populations of interest. Parameter values for *p_1_* and *a* are shown above each plot.

We designed a metagenomic simulation study in which ten subjects are drawn from two groups - the sampling depth of each subject was determined by random sampling from a uniform distribution between 200 and 1000 (these depths are reasonable for metagenomic studies). Given a population mean proportion *p* and a dispersion value *φ*, we sample sequences from a beta-binomial distribution *Β*(*α,β*), where *α* = *p*(1/*φ*−1) and *β* = (1−*p*)(1/*φ*−1). Note that data from this sampling procedure fits the assumptions for Lu *et al.* as well as Robinson and Smyth and therefore we expect them to do well under these conditions. Lu *et al.* designed a similar study for SAGE data, however, for each simulation, a fixed dispersion was used for both populations and the dispersion estimates were remarkably small (*φ* = 0, 8e-06, 2e-05, 4.3e-05). Though these values may be reasonable for SAGE data, we found that they do not accurately model metagenomic data. Figure 3 displays estimated dispersions within each population for all features of the metagenomic datasets examined below. Dispersion estimates range from 1e-07 to 0.17, and rarely do the two populations share a common dispersion. Thus we designed our simulation so that *φ* is chosen for each population randomly from a uniform distribution between 1e-08 and 0.05, allowing for potential significant differences between population distributions. For each set of parameters, we simulated 1000 feature counts, 500 of which are generated under *p_1_* = *p_2_*, the remainder are differentially abundant where a*p_1_ = *p_2_*, and compared the performance of each method using receiver-operating-characteristic (ROC) curves. Figure 4 displays the ROC results for a range of values for *p* and *a*. For each set of parameters, Metastats was run using 5000 permutations to compute p-values. Metastats performs as well as other methods, and in some cases is preferable. We also found that in most cases our method was more sensitive than the negative binomial model, which performed poorly for high abundance features.

**Figure 5:**
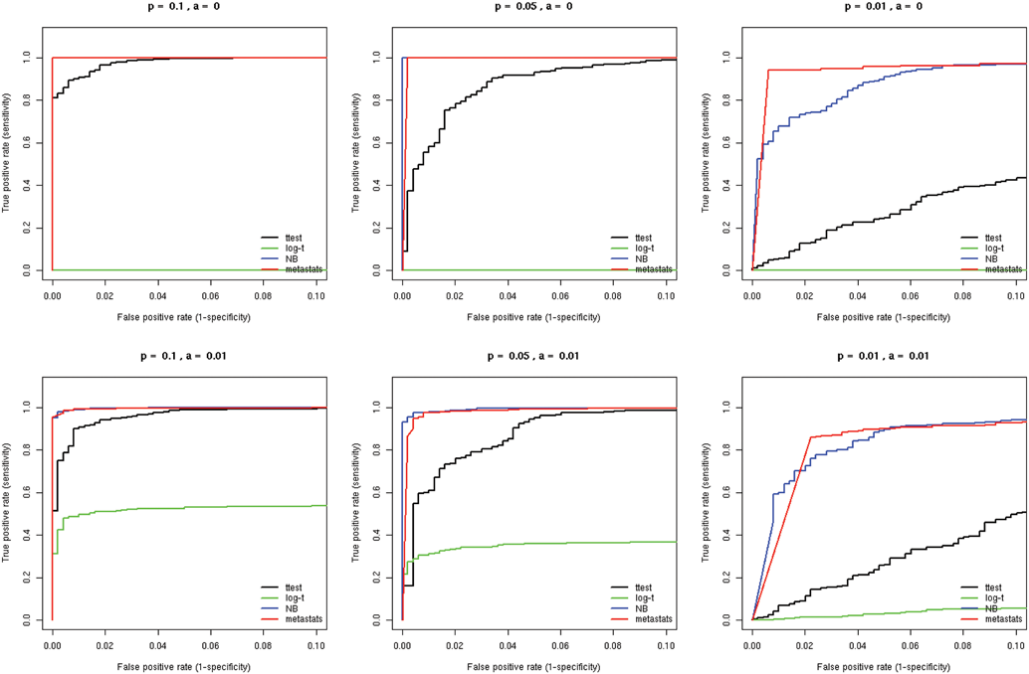
ROC curves comparing statistical methods in a simulation study for extreme sparse sampling. Sequences were selected from a beta-binomial distribution with variable dispersions and group mean proportions *p_1_* and *p_2_*. For each set of parameters, we simulated 1000 trials, 500 of which are generated under the null hypothesis (*p_1_* = *p_2_*), and the remainder are differentially abundant where a*p_1_ = *p_2_*. For example, p = 0.2 and a = 2 indicates features comprising 20% of the population that differ two-fold in abundance between two populations of interest. Parameter values for *p_1_* and *a* are shown above each plot.

Our next simulation sought to examine the accuracy of each method under extreme sparse sampling. As shown in the datasets below, it is often the case that a feature may not have any observations in one population, and so it is essential to employ a statistical method that can address this frequent characteristic of metagenomic data. Under the same assumptions as the simulation above, we tested *a* = 0 and 0.01, thereby significantly reducing observations of a feature in one of the populations. The ROC curves presented in Figure 5 reveal that Metastats outperforms other statistical methods in the face of extreme sparseness. Holding the false positive rate (x-axis) constant, Metastats shows increased sensitivity over all other methods. The poor performance of Log-t is noteworthy given it is designed for SAGE data that is also potentially sparse. Further investigation revealed that the Log-t method results in a highly inflated dispersion value if there are no observations in one population, thereby reducing the estimated significance of the test.

Finally, we selected a subset of the Dinsdale *et al.* [6] metagenomic subsystem data (described below), and randomly assigned each subject to one of two populations (20 subjects per population). All subjects were actually from the same population (microbial metagenomes), thus the null hypothesis is true for each feature tested (no feature is differentially abundant). We ran each methodology on this data, recording computed p-values for each feature. Repeating this procedure 200 times, we simulated tests of 5200 null features. Table 1 displays the number of false positives incurred by each methodology given different p-value thresholds. The results indicate that the negative binomial model results in an exceptionally high number of false positives relative to the other methodologies. Student's *t*-test and Metastats perform equally well in estimating the significance of these null features, while Log-t performs slightly better.

**Table 1:** Comparison of false positives found by different methodologies. Using real metagenomic data, we simulated features with no differential abundance by randomly dividing subjects from a single population into two subpopulations.

P≤	Number of False Positives			
	Metastats	Student-t	Log-t	NB
0.001	7	4	4	109
0.005	25	25	24	121
0.01	51	52	43	133

We found that for a stringent p-value threshold of 0.001, the negative binomial model (NB) resulted in a false positive rate 20 times higher than the other methodologies. The Log-t of Lu *et al.* resulted in the lowest false positive rate among the methods tested while Student's test and Metastats performed equally well.

These studies show that Metastats consistently performs as well as all other applicable methodologies for deeply-sampled features, and outperforms these methodologies on sparse data. Below we further evaluate the performance of Metastats on several real metagenomic datasets.

### Taxa associated with human obesity

In a recent study, Ley *et al.* [35] identified gut microbes associated with obesity in humans and concluded that obesity has a microbial element, specifically that Firmicutes and Bacteroidetes are bacterial divisions differentially abundant between lean and obese humans. Obese subjects had a significantly higher relative abundance of Firmicutes and a lower relative abundance of Bacteriodetes than the lean subjects. Furthermore, obese subjects were placed on a calorie-restricted diet for one year, after which the subjects' gut microbiota more closely resembled that of the lean individuals.

We obtained the 20,609 16S rRNA genes sequenced in Ley *et al.* and assigned them to taxa at different levels of resolution (note that 2,261 of the 16S sequences came from a previous study [36]). We initially sought to re-establish the primary result from this paper using our methodology. Table 2 illustrates that our method agreed with the results of the original study: Firmicutes are significantly more abundant in obese subjects (P = 0.003) and Bacteroidetes are significantly more abundant in the lean population (P<0.001). Furthermore, our method also detected Actinobacteria to be differentially abundant, a result not reported by the original study. Approximately 5% of the sample was composed of Actinobacteria in obese subjects and was significantly less frequent in lean subjects (P = 0.004). *Collinsella* and *Eggerthella* were the most prevalent Actinobacterial genera observed, both of which were overabundant in obese subjects. These organisms are known to ferment sugars into various fatty acids [37], further strengthening a possible connection to obesity. Note that the original study used Students *t*-test, leading to a p-value for the observed difference within Actinobacteria of 0.037, 9 times larger than our calculation. This highlights the sensitivity of our method and explains why this difference was not originally detected.

**Table 2:** Differentially abundant taxa between lean and obese human gut microflora.

Taxon	Obese abundance(%)	Lean abundance(%)	Metastats p-value	Student-t p-value	Log-t p-value	NB p-value
**Phyla**						
Bacteroidetes	2.902±1.067	25.652±4.576	0.0002	0.0000	0.0004	0.0000
Firmicutes	89.318±2.223	72.833±4.812	0.0028	0.0025	0.0030	0.8701
Actinobacteria	4.490±1.345	0.447±0.179	0.0037	0.0371	0.0004	0.0773
**Classes**						
Bacteroidetes (class)	2.722±1.065	25.652±4.576	0.0001	0.0000	0.0005	0.0001
Actinobacteria (class)	4.490±1.345	0.447±0.179	0.0024	0.0371	0.0004	0.1858
Clostridia	84.633±2.388	66.907±5.799	0.0036	0.0042	0.0052	0.9797
**Genera**						
*Syntrophococcus*	2.380±0.383	0.666±0.337	0.0014	0.0077	0.0067	0.4860
*Terasakiella*	0.000±0	0.115±0.115	0.0016	0.1986	0.9963	0.0166
*Ruminococcus*	26.276±4.454	10.707±2.094	0.0023	0.0207	0.0039	0.6639
*Marinilabilia*	0.010±0.010	0.138±0.138	0.0024	0.2353	0.0467	0.0011
*Collinsella*	3.565±1.187	0.154±0.154	0.0052	0.0451	0.0046	0.6545
*Bacteroides*	1.841±0.963	14.623±4.444	0.0056	0.0023	0.0105	0.0012
*Paludibacter*	0.000±0	0.093±0.069	0.0059	0.0896	0.9963	0.0000
*Bryantella*	0.461±0.051	0.151±0.102	0.0065	0.0072	0.0304	0.0487
*Desulfovibrio*	0.031±0.031	0.145±0.145	0.0073	0.3390	0.2315	0.0156

For the phylum, class, and genus levels (mean percentage±s.e., p-value≤0.01) we successfully re-established the major result of Ley *et al.*, and uncovered a new difference within Actinobacteria. Both Firmicutes and Actinobacteria have significantly higher relative abundances in obese people, while Bacteroidetes make up a higher proportion of the gut microbiota in the lean population. Results reveal Clostridia as the primary component of the differential abundance observed within Firmicutes, and Bacteroidetes and Actinobacteria classes explain the differential abundance observed within the corresponding phyla. Using this p-value threshold, we expect less than one false positive among these results. The last four columns display the computed p-values for different statistical methods, including Metastats and the overdispersion methods of Lu *et al.* (Log-t) and Robinson and Smyth (NB). These results reveal NB and Student's *t*-test to be overly-conservative.

To explore whether we could refine the broad conclusions of the initial study, we re-analyzed the data at more detailed taxonomic levels. We identified three classes of organisms that were differentially abundant: Clostridia (P = 0.005), Bacteroidetes (P<0.001), and Actinobacteria (P = 0.003). These three were the dominant members of the corresponding phyla (Firmicutes, Bacteroides, Actinobacteria, respectively) and followed the same distribution as observed at a coarser level. Metastats also detected nine differentially abundant genera accounting for more than 25% of the 16S sequences sampled in both populations (P≤0.01). *Syntrophococcus*, *Ruminococcus*, and *Collinsella* were all enriched in obese subjects, while *Bacteroides* on average were eight times more abundant in lean subjects.

For taxa with several observations in each subject, we found good concordance between our results (p-value estimates) and those obtained with most of the other methods (Table 2). Surprisingly, we found that the negative binomial model of Robinson and Smyth failed to detect several strongly differentially abundant features in these datasets (e.g the hypothesis test for Firmicutes results in a p-value of 0.87). This may be due in part to difficulties in estimating the parameters of their model for our datasets and further strengthens the case for the design of methods specifically tuned to the characteristics of metagenomic data. For cases where a particular taxon had no observations in one population (e.g. *Terasakiella*), the methods proposed for SAGE data seem to perform poorly.

### Differentially abundant COGs between mature and infant human gut microbiomes

Targeted sequencing of the 16S rRNA can only provide an overview of the diversity within a microbial community but cannot provide any information about the functional roles of members of this community. Random shotgun sequencing of environments can provide a glimpse at the functional complexity encoded in the genes of organisms within the environment. One method for defining the functional capacity of an environment is to map shotgun sequences to homologous sequences with known function. This strategy was used by Kurokawa *et al.* [34] to identify clusters of orthologous groups (COGs) in the gut microbiomes of 13 individuals, including four unweaned infants. We examined the COGs determined by this study across all subjects and used Metastats to discover differentially abundant COGs between infants and mature (>1 year old) gut microbiomes. This is the first direct comparison of these two populations as the original study only compared each population to a reference database to find enriched gene sets. Due to the high number of features (3868 COGs) tested for this dataset and the limited number of infant subjects available, our method used the pooling option to compute p-values (we chose 100 permutations), and subsequently computed q-values for each feature. Using a threshold of Q≤0.05 (controlling the false discovery rate to 5%), we detected 192 COGs that were differentially abundant between these two populations (see Table 3 for a lisitng of the most abundant COGs in both mature and infant microbiomes. Full results are presented as supplementary material in Table S1).

**Table 3:** COGs determined to be differentially abundant between infant and mature human gut microbiomes using a q-value threshold of 0.05.

COG id	Description	Mature		Infant		Metastat q-value
		Mean abundance (%)	stderr	Mean abundance (%)	stderr	
COG0205	6-phosphofructokinase	0.0017	0.0001	0.0006	0.0002	0.0313
COG0358	DNA primase (bacterial type)	0.0024	0.0001	0.0008	0.0001	0.0072
COG0507	ATP-dependent exoDNAse (exonuclease V), alpha subunit - helicase superfamily I member	0.0016	0.0001	0.0008	0.0001	0.0349
COG0526	Thiol-disulfide isomerase and thioredoxins	0.0028	0.0002	0.0014	0.0002	0.0371
COG0621	2-methylthioadenine synthetase	0.0017	0.0001	0.0008	0.0002	0.0450
COG0642	Signal transduction histidine kinase	0.0132	0.0009	0.0070	0.0004	0.0270
COG0667	Predicted oxidoreductases (related to aryl-alcohol dehydrogenases)	0.0012	0.0001	0.0021	0.0001	0.0282
COG0739	Membrane proteins related to metalloendopeptidases	0.0024	0.0001	0.0006	0.0001	0.0072
COG0745	Response regulators consisting of a CheY-like receiver domain and a winged-helix DNA-binding domain	0.0076	0.0003	0.0051	0.0004	0.0352
COG0747	ABC-type dipeptide transport system, periplasmic component	0.0011	0.0001	0.0027	0.0003	0.0352
COG1113	Gamma-aminobutyrate permease and related permeases	0.0002	0.0001	0.0018	0.0003	0.0349
COG1129	ABC-type sugar transport system, ATPase component	0.0013	0.0001	0.0028	0.0003	0.0492
COG1145	Ferredoxin	0.0017	0.0001	0.0005	0.0002	0.0217
COG1196	Chromosome segregation ATPases	0.0017	0.0001	0.0007	0.0001	0.0108
COG1249	Pyruvate/2-oxoglutarate dehydrogenase complex, dihydrolipoamide dehydrogenase (E3) component, and related enzymes	0.0006	0.0001	0.0011	0.0001	0.0349
COG1263	Phosphotransferase system IIC components, glucose/maltose/N-acetylglucosamine-specific	0.0012	0.0001	0.0031	0.0003	0.0313
COG1475	Predicted transcriptional regulators	0.0025	0.0002	0.0014	0.0002	0.0435
COG1595	DNA-directed RNA polymerase specialized sigma subunit, sigma24 homolog	0.0053	0.0004	0.0013	0.0003	0.0206
COG1609	Transcriptional regulators	0.0030	0.0002	0.0092	0.0013	0.0424
COG1629	Outer membrane receptor proteins, mostly Fe transport	0.0120	0.0016	0.0013	0.0007	0.0313
COG1762	Phosphotransferase system mannitol/fructose-specific IIA domain (Ntr-type)	0.0004	0.0001	0.0017	0.0002	0.0293
COG1961	Site-specific recombinases, DNA invertase Pin homologs	0.0059	0.0004	0.0018	0.0006	0.0345
COG2204	Response regulator containing CheY-like receiver, AAA-type ATPase, and DNA-binding domains	0.0019	0.0002	0.0005	0.0002	0.0421
COG2244	Membrane protein involved in the export of O-antigen and teichoic acid	0.0019	0.0001	0.0009	0.0001	0.0229
COG2376	Dihydroxyacetone kinase	0.0002	0	0.0009	0.0001	0.0278
COG2440	Ferredoxin-like protein	0	0	0.0002	0	0.0394
COG2893	Phosphotransferase system, mannose/fructose-specific component IIA	0.0003	0.0001	0.0011	0.0001	0.0352
COG3250	Beta-galactosidase/beta-glucuronidase	0.0056	0.0004	0.0023	0.0006	0.0435
COG3451	Type IV secretory pathway, VirB4 components	0.0033	0.0001	0.0009	0.0003	0.0157
COG3505	Type IV secretory pathway, VirD4 components	0.0029	0.0001	0.0010	0.0003	0.0278
COG3525	N-acetyl-beta-hexosaminidase	0.0016	0.0002	0.0004	0.0001	0.0352
COG3537	Putative alpha-1,2-mannosidase	0.0020	0.0003	0.0002	0.0002	0.0352
COG3711	Transcriptional antiterminator	0.0004	0.0001	0.0020	0.0003	0.0339
COG3712	Fe2+-dicitrate sensor, membrane component	0.0023	0.0003	0	0	0.0280
COG4206	Outer membrane cobalamin receptor protein	0.0021	0.0003	0.0003	0.0001	0.0313
COG4771	Outer membrane receptor for ferrienterochelin and colicins	0.0039	0.0005	0.0006	0.0003	0.0366

Using this threshold we expect less than 10 false positives in this data-set. The table presents the 25 most abundant COGs from the mature and infant microbiomes, sorted by their abundance level in the mature population. Full results are available as supplementary material.

The most abundant enriched COGs in mature subjects included signal transduction histidine kinase (COG0642), outer membrane receptor proteins, such as Fe transport (COG1629), and Beta-galactosidase/beta-glucuronidase (COG3250). These COGs were also quite abundant in infants, but depleted relative to mature subjects. Infants maintained enriched COGs related to sugar transport systems (COG1129) and transcriptional regulation (COG1475). This over-abundance of sugar transport functions was also found in the original study, strengthening the hypothesis that the unweaned infant gut microbiome is specifically designed for the digestion of simple sugars found in breast milk. Similarly, the depletion of Fe transport proteins in infants may be associated with the low concentration of iron in breast milk relative to cow's milk [38]. Despite this low concentration, infant absorption of iron from breast milk is remarkably high, and becomes poorer when infants are weaned, indicating an alternative mechanism for uptake of this mineral. The potential for a different mechanism is supported by the detection of a Ferredoxin-like protein (COG2440) that was 11 times more abundant in infants than in mature subjects, while Ferredoxin (COG1145) was significantly enriched in mature subjects.

### Differentially abundant metabolic subsystems in microbial and viral metagenomes

A recent study by Dinsdale *et al.* profiled 87 different metagenomic shotgun samples (∼15 million sequences) using the SEED platform (http://www.theseed.org) [6] to see if biogeochemical conditions correlate with metagenome characteristics. We obtained functional profiles from 45 microbial and 40 viral metagenomes analyzed in this study. Within the 26 subsystems (abstract functional roles) analyzed in the Dinsdale *et al.* study, we found 13 to be significantly different (P≤0.05) between the microbial and viral samples (Table 4). Subsystems for RNA and DNA metabolism were significantly more abundant in viral metagenomes, while nitrogen metabolism, membrane transport, and carbohydrates were all enriched in microbial communities. The high levels of RNA and DNA metabolism in viral metagenomes illustrate their need for a self-sufficient source of nucleotides. Though the differences described by the original study did not include estimates of significance, our results largely agreed with the authors' qualitative conclusions. However, due to the continuously updated annotations in the SEED database since the initial publication, we found several differences between our results and those originally reported. In particular we found virulence subsystems to be less abundant overall than previously reported, and could not find any significant differences in their abundance between the microbial and viral metagenomes.

**Table 4:** Differentially abundant metabolic subsystems between microbial and viral metagenomes (mean percentage±s.e., p-values≤0.05).

Subsystem	microbial	viral	Metastats p value
Carbohydrates	17.01±0.77	12.87±0.82	0.001
Amino Acids and Derivatives	9.29±0.46	7.58±0.55	0.019
Respiration	8.24±1.34	3.89±0.46	0.001
Photosynthesis	7.13±2.38	1.16±0.36	0.017
Cofactors, Vitamins, and Pigments	5.54±0.27	6.44±0.26	0.022
Experimental Subsystems	4.88±0.31	5.80±0.36	0.050
DNA Metabolism	3.99±0.24	9.18±1.06	0.001
Cell Wall and Capsule	3.73±0.27	5.64±0.71	0.009
RNA Metabolism	3.65±0.21	5.23±0.71	0.033
Nucleosides and Nucleotides	3.38±0.18	7.72±0.74	0.001
Membrane Transport	2.04±0.11	1.30±0.15	0.001
Nitrogen Metabolism	1.47±0.08	0.93±0.10	0.001
Fatty Acids and Lipids	1.46±0.07	1.05±0.11	0.004

Using this threshold we expect less than one false positive in the data-set. We find that viral metagenomes are significantly enriched for nucleotides and nucleosides and DNA metabolism, consistent with the viruses' need for self-sufficiency. Processes for respiration, photosynthesis, and carbohydrates are overrepresented in microbial metagenomes.

## Discussion

We have presented a statistical method for handling frequency data to detect differentially abundant features between two populations. This method can be applied to the analysis of any count data generated through molecular methods, including random shotgun sequencing of environmental samples, targeted sequencing of specific genes in a metagenomic sample, digital gene expression surveys (e.g. SAGE [29]), or even whole-genome shotgun data (e.g. comparing the depth of sequencing coverage across assembled genes). Comparisons on both simulated and real dataset indicate that the performance of our software is comparable to other statistical approaches when applied to well- sampled datasets, and outperforms these methods on sparse data.

Our method can also be generalized to experiments with more than two populations by substituting the *t*-test with a one-way ANOVA test. Furthermore, if only a single sample from each treatment is available, a chi-squared test can be used instead of the *t*-test. [27].

In the coming years metagenomic studies will increasingly be applied in a clinical setting, requiring new algorithms and software tools to be developed that can exploit data from hundreds to thousands of patients. The methods described above represent an initial step in this direction by providing a robust and rigorous statistical method for identifying organisms and other features whose differential abundance correlates with disease. These methods, associated source code, and a web interface to our tools are freely available at http://metastats.cbcb.umd.edu.

## Supporting Information

Table S1 Comparison of COG composition between mature and infant microbiomes. The relative abundance of each COG is presented together with significance values (likelihood that difference in abundance is due to chance alone)computed using several statistical procedure: metastats (p and q values), student's t-test, log linear model, and negative binomial model.
(0.95 MB XLS)
